# Routine gastric residual volume measurement and energy target achievement in the PICU: a comparison study

**DOI:** 10.1007/s00431-017-3015-8

**Published:** 2017-09-18

**Authors:** Lyvonne N. Tume, Anna Bickerdike, Lynne Latten, Simon Davies, Madeleine H. Lefèvre, Gaëlle W. Nicolas, Frédéric V. Valla

**Affiliations:** 1 Faculty of Health and Applied Sciences, University of West of England, Glenside Campus, Blackberry Hill, Stapleton, Bristol, BS16 1DD UK; 2 School of Medicine, University of Liverpool, MBChB Office, Cedar House, Ashton Street, Liverpool, L69 3GE UK; 3 Department of Dietetics, Alder Hey Children’s NHS Foundation Trust, Eaton Road, Liverpool, L12 2AP UK; 4 Department of Anaesthesia, Royal Oldham Hospital, Rochdale Road, Manchester, OL1 2JH UK; 5 University of Lyon Claude Bernard Lyon 1, 43 Boulevard du 11 Novembre 1918, 69100 Lyon-, Villeurbanne, France; 6 Pediatric Intensive Care Unit, Hôpital Femme Mère Enfant, Hospices Civils de Lyon, 59 bd Pinel, 69500 Lyon-, Bron, France

**Keywords:** Enteral feeding, Paediatric intensive care, Nursing practice, Nutrition, Feeding tolerance

## Abstract

Critically ill children frequently fail to achieve adequate energy intake, and some care practices, such as the measurement of gastric residual volume (GRV), may contribute to this problem. We compared outcomes in two similar European Paediatric Intensive Care Units (PICUs): one which routinely measures GRV (PICU-GRV) to one unit that does not (PICU-noGRV). An observational pilot comparison study was undertaken. Eighty-seven children were included in the study, 42 (PICU-GRV) and 45 (PICU-noGRV). There were no significant differences in the percentage of energy targets achieved in the first 4 days of PICU admission although PICU-noGRV showed more consistent delivery of median (and IQR) energy targets and less under and over feeding for PICU-GRV and PICU-noGRV: day 1 37 (14–72) vs 44 (0–100), day 2 97 (53–126) vs 100 (100–100), day 3 84 (45–112) vs 100 (100–100) and day 4 101 (63–124) vs 100 (100–100). The incidence of vomiting was higher in PICU-GRV. No necrotising enterocolitis was confirmed in either unit, and ventilator-acquired pneumonia rates were not significantly different (7.01 vs 12 5.31 per 1000 ventilator days; *p* = 0.70) between PICU-GRV and PICU-noGRV units.

*Conclusions*: The practice of routine gastric residual measurement did not significantly impair energy targets in the first 4 days of PICU admission. However, not measuring GRV did not increase vomiting, ventilator-acquired pneumonia or necrotising enterocolitis, which is the main reason clinicians cite for measuring GRV.

**Table:** 

**What is known:** • *The practice of routinely measuring gastric residual volume is widespread in critical care units* • *This practice is increasingly being questioned in critically ill patients, both as a practice that increases* • *The likelihood of delivering inadequate enteral nutrition amounts and as a tool to assess feeding tolerance*
**What is new:** • *Not routinely measuring gastric residual volume did not increase adverse events of ventilator acquired pneumonia, necrotising enterocolitis or vomiting.* • *In the first 4 days of PICU stay, energy target achievement was not significantly different, but the rates of under and over feeding were higher in the routine GRV measurement unit.*

## Introduction

Inadequate delivery of enteral nutrition remains a problem in critically ill children. An international study involving 800 children in 31 PICUs found that only 37% of children received their prescribed energy intake whilst in intensive care, and it took nearly 12 days for them to achieve even 90% of their calorie target [23]. A common nursing practice to assess enteral nutrition (EN) ‘tolerance’ is to measure gastric residual volume (GRV) regularly in critically ill patients, and it is often a factor in the decision to stop or hold enteral nutrition [33, 35]. Indeed, perceived ‘high’ GRV levels often lead to withholding EN, and such interruptions are a common barrier to delivering EN in PICUs [16]. Despite this, the evidence for GRV to assess feed tolerance is poor, with GRV not correlating consistently to enteral feeding volumes and the measurement itself often being inaccurate [2, 18, 20]. In addition, what volume constitutes an ‘acceptable’ level of GRV remains unknown. GRV is routinely measured in all UK PICUs [33]. This practice, however, is very variable in terms of frequency, acceptable volumes and actions in response to GRV [33], yet it is not standard practice in 40% of French PICUs [35]. Thus, we aimed to compare outcomes in a PICU which routinely measures GRV to a PICU that does not.

## Methods

An observational pilot comparison study was undertaken between two paediatric intensive care units. PICU-GRV is a PICU that routinely measured GRV in Liverpool, UK; PICU-noGRV is a unit that does not routinely measure GRV in Lyon, France. The units were comparable by size and volume, but as the PICU-noGRV did not admit cardiac surgical children, these patients were excluded in PICU-GRV. The study objectives were to identify whether routine GRV measurement impacted on energy delivery in mechanically ventilated PICU patients and to identify whether routine GRV measurement impacted on the incidence of complications: vomiting, necrotising enterocolitis (NEC) and ventilator-acquired pneumonia (VAP). The study inclusion criteria were as follows:

- Mechanically ventilated children (0–17 years) admitted onto the PICU with a nasogastric tube or gastrostomy tube in situ
- Invasive ventilation expected to last for more than 72 h

The exclusion criteria were as follows:

- Post-operative cardiac surgical children
- Pre-term infants < 37 weeks’ gestation (but history of prematurity was not an exclusion criteria)
- Children > 17 years of age
- Children who had contraindications for enteral feeding according to local guidelines (see Table 1)
- Children who received post-pyloric feeding

**Table 1:** Comparison of standard practices in study units that may have an impact on feeding tolerance and gastric clearance

Variable/practice	PICU 1 (routine GRV)	PICU 2 (no routine GRV)
Unit Size	24 PICU beds Admits 0–17 years	23 PICU and HDU beds Admits 0–17 years
RN: patient ratio	1:1	1:2
Nursing staff with specialist PIC qualification	52%	5%
Written feeding policy	Yes	Yes
Dedicated dietetic support	Yes	Yes
Energy target estimation	Schofield equation (adjusted age, sex weight)	Schofield equation—adjusted age, sex, height, weight) or (Schofield + RDA) / 2 in infants < 4 months
Energy goals (sedated ventilated children)	Aims to achieve target predicted energy requirements by day 2 or 3	Aims to achieve target predicted energy requirements by day 2 or 4
GRV measured	Yes, every 4–5 h Feeds withheld if > 5 mls/kg or maximum 300 ml	No
Feeding method used	Bolus feeding in infants q 2–3 hourly and continuous feeds (4 h on 1 h off) in older children, but method is RN decision	All continuous over 24 h continuous infusion rate calculated on a 23 h basis (mL/h = daily prescribed volume/23) to compensate involuntary delay
Target feed start time	Within 6 h of admission	Within 24 h of admission
Feed advancement rate	Dependant on feed tolerance based on the GRV measurements above	Once a day with the aim to meet energy targets within 48–96 h
Jejunal tubes	Not as a first line, except severe burns, but placed if NG feeds not tolerated	Not in the first place; if high risk patients (brain injury) or feeding intolerance
Polymeric/semi-elemental feeds	Polymeric unless history of short gut/liver dysfunction	Polymeric unless child on elemental feeds prior to admission
Isocaloric/hyper-caloric feeds	Isocaloric first line then adjusted to meet requirements	Isocaloric (85% patients)
Fibres/no fibres in EN	Fibre feeds except if history of GI pathology (short gut, etc)	Fibres added
Use of prokinetics, laxatives	Not routine, only in traumatic brain injury or if feeding problems	Not routine, only in acute neurological disease or if feeding problems
Guidance on withholding EN	Bowel obstruction, active gut haemorrhage, non-intubated patients with acute altered consciousness Not restricted if on vasoactive drugs or, only if serum lactate > 2 mmol/l	Bowel obstruction, active gut haemorrhage, non-intubated patients with acute altered consciousness, increasing doses of vasoactive drugs but physician-dependent
Guidance on stopping EN	Vomiting, abdominal distension, pain If lactate > 2 mmol/l, large GRV > 5 ml/kg or 300 ml 6 h prior to extubation For transport: depends on procedure	Vomiting, abdominal distension, pain 4 h prior to extubation For transport: immediately prior
Use of cuffed ETTs	75%	95%
Usual sedation and analgesia for > 1 day ventilation	Morphine or fentanyl and midazolam	Sufentanyl and midazolam ± ketamine
Sedation assessment score	COMFORT-B	COMFORT-B
VAP bundle	Yes, with head up and regular oral care and closed suction	No but all nursed head up 30–45 degrees + regular oral care
Use of neuromuscular blocking agents	30% patients (mainly brain injury, unstable airways, difficult ventilation ARDS)	7% of all patients (mainly ARDS, brain injury, airway surgery)
VAP diagnostic criteria	CDC 2009 and antibiotics started	CDC 2009 and antibiotics started

Shaded rows indicate major differences between PICU-GRV and PICU noGRV

*RN* registered nurse, *PIC* Paediatric Intensive Care, *GRV* gastric residual volume, *HDU* high dependency unit, *EN* enteral nutrition, *ETTs* endotracheal tubes, *COMFORT-B* a sedation scoring tool, *VAP* ventilator acquired pneumonia, *ARDS* acute respiratory distress syndrome, *RDA* recommended dietary allowance, *CDC* Centre for Disease Control, *NG* nasogastric

Data was collected in 2016–2017 prospectively in PICU-GRV and retrospectively in PICU-noGRV at two time points within a 12-month period. The settings and standard practices (that may impact on nutrition and enteral feeding) are compared between the two centres in Table 1. Both units used predictive equations (Schofield equation) to set energy targets in ventilated children, as neither centre used indirect calorimetry. PICU-noGRV increased energy targets for children under the age of 4 months, following the equation (Schofield + RDA)/2.

The primary outcome used in this study was the percentage of target energy requirements achieved per day of the child’s PICU stay. Secondary outcomes were incidence of vomiting, NEC (in infants) and VAP. VAP was defined consistently using the 2009 CDC criteria. The diagnosis of NEC was confirmed using Bell’s criteria and suspected NEC defined if active intervention was taken (instituting fasting and the commencement of triple antibiotics). Vomiting was defined pragmatically, as that reported and documented by the bedside nurse and any incidence of these per 24 h was considered as a positive event.

## Data analysis

Data collected was entered into a Microsoft Excel database. Descriptive statistics were undertaken first. Normally distributed data is presented as mean (SD) and non-normally distributed data as median and IQR. The data was then imported into IBM SPSS version 22 for further inferential analysis. Inferential analysis undertaken compared the two groups. Independent *t* test was used if the data was normally distributed and non-parametric tests, Mann-Whitney and Chi-square used if it was not. A *p* value < 0.05 was considered significant and two-tailed tests were used. The UK study was registered as service evaluation with the NHS Trust (reference 5194) and in France, the centre received IRB approval (Reference No. 00009118, Comité de protection des personnes 89 Lyon sud-est 2); in both instances, a waiver of consent was granted as no identifiable patient data was collected.

## Results

Eighty-seven children who met the study inclusion criteria were included in the study over two time periods in a 12-month period: 42 in PICU-GRV and 45 in PICU NoGRV. There was no significant difference in age, weight or sex between the two groups. The median age was 5.3 months (PICU-GRV) vs 9.7 months (PICU-noGRV); the median weight was 5.4 kg (PICU-GRV) vs 9.8 kg (PICU-noGRV) and 60% children were male (Table 2). The majority of admitted children had a medical diagnosis, mostly respiratory or neurological failure (Table 2). Children in PICU-noGRV were significantly sicker at admission (*p* < 0.001) and had significantly longer length of ventilation (*p* = < 0.001) and length of PICU stay (*p* = < 0.001). Between PICU-GRV and PICU-noGRV, a comparison of standard practices that may have an impact on feeding tolerance and gastric clearance was broadly similar. (Table 1). The main differences were that PICU-GRV initiated enteral feeding significantly earlier than did PICU noGRV (mean 7.8 (7.4) versus 21.5 h (18.3)) (*p* = <0.001), and PICU-noGRV fed all (100%) children continuously, compared to 41% in PICU-GRV (*p* = < 0.001). There were no significant differences in the median percentage of energy targets achieved in the first 4 days of PICU admission; however, PICU-noGRV showed more consistent (with less variance around 100% of the predicted energy targets achieved) and less under and over feeding (Table 3). The incidence of vomiting (between day 1 to 4) was higher in the PICU-GRV but was not statistically significant (*p* = 0.339). No NEC was confirmed in either centre and VAP rates were similar 7.01 per 1000 ventilator days (2/42) in PICU-GRV and 5.31 per 1000 ventilator days (3/45) in PICU-noGRV (*p* = 0.70), despite the significantly longer length of ventilation in PICU-noGRV (Table 4).

**Table 2:** Patient demographics

Demographic	PICU-GRV *N* = 42	PICU-noGRV *N* = 45	*P* value
Age (months)			
median (IQR)	5.3 (1.9–44.5)	9.7 (1.5–78)	0.724
Sex (% male)	61.9% (26/42)	57.7% (26/45)	0.412
Admission weight (kg)			
Median (IQR)	5.4 (3.8–15.5)	9.8 (4.09–26)	0.220
Z score (weight for age) (mean SD)	0.043 (1.06)	0.104 (1.60)	0.834
Diagnostic group/PICU admission reason	Respiratory failure 81%	Respiratory failure 42%	
	Neurological failure 10%	Neurological failure 42%	
	Sepsis 4%	Sepsis 6.6%	
	Cardiovascular 2%	Cardiovascular 4.4%	
	Miscellaneous 3%	Post-op surgical 2.2%	
		Trauma 2.2%	
		Miscellaneous 0.6%	
PIM2 score			
Mean (SD)	0.05 (0.079)	0.09 (.218)	< 0.001
No IV sedation/opiate	21% (9/42)	0%	< 0.001
IV Opiate ± sedation	50% (21/42)	58% 26/46	
Sedation + neuromuscular blockade	29% (12/42)	42% (19/45)	0.263
Died	2.3% (1/42)	6.6% (3/45)	0.339
LOV (days)			
Median (IQR)	5 (3–7)	7.5 (5.9–11.7)	< 0.001
LOS (days)			
Median (IQR)	6 (3–9)	13 (11–20)	< 0.001

*PICU* Paediatric Intensive Care Unit, *GRV* gastric residual volume, *IQR* interquartile range, *PIM2* a paediatric risk of mortality scoring tool, *SD* standard deviation, *LOV* length of mechanical ventilation, *LOS* length of PICU stay

**Table 3:** Detailed enteral feeding data per PICU

EN parameter	PICU-GRV *n* = 42	PICU-noGRV *n* = 45	*P* value
Time to first feed (hours) mean and SD	7.84 (7.38)	21.5 (18.3)	< 0.001
Percentage of children continuously fed	41% (17/41)	100% (45/45)	< 0.001
Percentage of energy prescribed actually delivered			
Day 1			
Mean (SD)	47.9 (41.1)	49.5 (49.9)	0.865
Median (IQR)	36.7 (14–72)	44.25 (0–100)	0.358
Day 2			
Mean (SD)	92.6 (52.2)	93.6 (44.6)	0.921
Median (IQR)	97 (52.8–126.2)	100 (99.6–100.8)	0.989
Day 3			
Mean (SD)	82.1 (40.3)	94.5 (22.5)	0.120
Median (IQR)	84.3 (45–112.5)	100 (100–100.5)	0.477
Day 4			
Mean (SD)	101.2 (39.2)	96.2 (16.9)	0.597
Median (IQR)	107 (63.1–124.2)	100 (100–100.8)	0.208
Daily hours no EN delivered			
Day 1	100% patients	2% patients	
Mean, (SD)	8.4 (5.3)	6 (SD 0)	
Day 2	100% patients	0% patients	
Mean (SD)	10.5 (6.2)		
Day 3	100% patients	6.6% patients	
Mean (SD)	10.1 (6.4)	14.3 (SD 4.4)	
Day 4	100% patients	8.8% patients	
Mean (SD)	9.6 (6.6)	8 (SD 2.7)	

*PICU* Paediatric Intensive Care Unit, *GRV* Gastric Residual Volume, *EN* enteral nutrition, *SD* standard deviation, *IQR* interquartile range

**Table 4:** Adverse events data outcomes

Vomiting incidence in first 4 days	PICU-GRV *n* = 42	PICU-noGRV *n* = 45	*P* value
Day 1 (n = 42)	7.1% (3/42)	4.4% (2/45)	
Day 2 (*n* = 39)	7.69% (3/39)	2.2% (1/45)	
Day 3 (*n* = 31)	3.2% (1/31)	0% (0/45)	
Day 4 (*n* = 20)	0% (0/20)	8.8% (4/45)	
Vomiting at any time (days 1–4)			0.39
Events during PICU admission			
VAP per 1000 ventilator days	7.1	5.3	0.70
Confirmed NEC	0	0	

*PICU* Paediatric Intensive Care Unit, *GRV* gastric residual volume, *VAP* ventilator acquired pneumonia, *NEC* necrotizing enterocolitis

In PICU-GRV, enteral feeding was withheld in all children for a median range of between 8.8–10.5 h a day in the first 4 days (Table 3). PICU-noGRV rarely withheld enteral feeding. In PICU-GRV, GRV was measured from 2 to 15 times in a 24-h period. The mean volume of aspirate obtained per patient was 1.2 mls/kg (median 0.96 mls/kg; range 0.03–3.3 mls/kg). Of the aspirates where fluid was aspirated, 77% of these were returned to the child and 23% were discarded, with the reason for this unclear.

## Discussion

This is the first study to our knowledge to attempt to describe the impact of routine GRV monitoring on critically ill children’s clinical outcomes. In two similar groups of patients, recruited in units with similar standards of nutritional care except for the measurement of GRV, we found that the routine measurement of GRV did not impair the achievement of energy goals in the first 4 days of PICU admission. More importantly, we showed that the practice was safe, with no difference in adverse events of VAP or NEC but with the incidence of vomiting higher in PICU-GRV. GRV measurement is based on many assumptions held by the healthcare team: the belief that the measurement is accurate, it represents gastric contents and helps to distinguish delayed gastric emptying; that high GRV only occurs if gastric emptying is delayed and indicates retention of enteral feed; the belief that increased volume of enteral feed in the stomach leads to vomiting and aspiration and that this aspiration leads to pneumonia (VAP) [18]. Indeed, as GRV is composed of both enteral feed and gastric secretions, it does not provide an accurate indicator of feed ‘tolerance’. No studies have ever demonstrated that measuring GRV reduces the risk of VAP, and no relationship has yet been established between higher gastric volumes and vomiting and pulmonary aspiration. Indeed, the measurement of GRV has been shown to frequently be inaccurate due to tube position in the stomach, syringe size, nasogastric tube diameter, feeding method and aspiration technique [2, 9, 19, 20]. Three adult intensive care trials, in predominantly medical patients, all found that not measuring GRV was safe and improved the achievement of energy targets [24, 27, 29]. More specifically, in these trials, accepting a higher GRV (500 ml compared to 200 ml) [24] or not measuring GRV at all [25, 27, 29] did not adversely affect patient outcomes of ventilator-associated pneumonia (VAP) or gastrointestinal complications; however, it did improve the achievement of energy goals. A further study showed that just by measuring GRV, the risk of delivering inadequate energy increased by 38% [28].

Increasingly, this routine practice is being challenged in neonatal intensive care [10, 17, 26] PICUs [33] and across critical care generally [4, 9, 15, 18, 30]. The incidence of gastrointestinal complication of vomiting was higher in the routine GRV measured group. We speculate that in this unit, the frequent withholding of enteral feeds has led to attempts to compensate afterwards in order to reach daily nutritional goals. This could have led to an increased EN infusion rate or volume, with increased vomiting. We had no NEC in either group; however, NEC is a disease of neonates affecting pre-term infants predominantly. As our cohort included few neonates, and pre-terms had been excluded, this was not surprising. The incidence of VAP (per 1000 ventilator days) was lower in PICU-noGRV (and non-significant) despite significantly higher PIM2 scores and longer length of ventilation that are risk factors for VAP in critically ill children [12]. The significantly higher severity of illness in PICU-noGRV might have been expected to negatively impact on energy delivery [5], but this was not the case. Despite a significantly longer time to initiate enteral feeding, once initiated, EN was tolerated well and was more consistently delivered in PICU-noGRV with sicker children.

In terms of the child’s achievement of their predicted energy targets, we were not able to compare data beyond day four, as there were too few ventilated children in PICU-GRV. It may be that as length of PICU stay increases, the impact of this practice on energy delivery may become more apparent, but we do not know this. Delivery of adequate nutrition and energy requirements to critically ill children is vital, and we know that these children frequently receive suboptimal nutrition [23], with many of them already malnourished at PICU admission [8]; this further adversely impacting on their clinical outcomes [11]. PICU-noGRV had more consistent achievement of estimated energy goals, with PICU-GRV demonstrating both under and overfeeding according to their respective local guidelines. On the one hand, GRV measurement in PICU-GRV seems to play a role in the higher incidence of underfeeding in this group. On the other hand, both units used predictive equations (Schofield equation) to set energy targets in ventilated children, as neither unit used indirect calorimetry [21, 31]. Indirect calorimetry, although the gold standard to guide energy targets in the critically ill [6, 7], is available in very few PICUs (14%) worldwide [14]. There are also accuracy imitations in the use of IC, mainly related to high-inspired oxygen requirements and air leaks, and predictive equations are considered acceptable in the absence of IC [21]. It is known that critically ill children need less energy than healthy children, and recommended dietary allowance (RDA) would provide too much energy in this setting. However, recent studies, comparing various equations, showed that Schofield equation, considered one of the most accurate, was less accurate in young infants, underestimating energy needs in the critically ill child [13]. As a consequence, PICU-noGRV local guidelines set higher energy targets than the one estimated by Schofield equation in infants younger than 4 months, defined as the mean between energy amounts calculated with Schofield equation and RDA (Schofield equation + RDA / 2). In contrast, PICU-GRV local guidelines used Schofield equation to set energy targets in all age groups (Table 1). Consequently, the same intake of energy afforded to this young age group, considered accurate in PICU-noGRV, would be considered as overnutrition in PICU-GRV, according to their respective guidelines. Bronchiolitis was the primary diagnosis in infants younger than 4 months, and considering that fluid allowance is less restrictive in these children (as they require less intravenous drugs than other patients), these patients were more likely to receive nutrition above Schofield equation estimation, thus being defined as overfed in PICU-GRV and normally fed in PICU-noGRV.

One of the main factors contributing to suboptimal energy delivery in the intensive care units is interruptions to enteral feeding [3, 16, 22]. In a recent survey of PICU nurses, elevated GRV was the main factor that led to feeds being withheld, but high GRVs have also been noted as a factor causing feed interruptions by others [1, 4, 15, 16, 22]. In our study, in the routine GRV measurement group, enteral feeds were withheld in all children for a median of 8 h in 24-h period, whereas in the no GRV group, enteral feeds were rarely withheld. Although interruptions may have occurred for other reasons (such as surgery or procedures) given the similarity in admitting diagnoses for both groups, this seems unlikely.

This pilot study has a number of limitations that need acknowledging. It was a pilot study (not informed by a power calculation), with small numbers, in only two European PICUs, and used both prospective and retrospective data collection which may have led to a patient selection bias. However, the retrospective data collection used a very detailed clinical information system with systematic record of nutritional intakes, vomiting and feeding intolerance symptoms. In addition, the incidence of NEC and VAP are very low overall, which make the results difficult to interpret. Despite our best efforts to ensure comparability of our patient populations and units, there may have been factors that affected our results. Our pragmatic definition of vomiting relied on nurse-reported data and may have been affected by other factors such as the child’s diagnosis or sedation level, leading to coughing which may contribute to vomiting, and we did not collect data on sedation level. We did collect data on diarrhoea but could not use this due to the difficulty in quantifying this; however, this is not the main factor that clinicians are concerned about regarding not measuring GRV. In PICU-GRV, there were significant feed stoppage times, despite the median GRVs being less than the unit guideline threshold. We did not collect data on the reason for these feed stoppages, nor with compliance with unit guidelines; thus, there may be other reasons (in addition to GRV) that affected our results. Furthermore, despite our best effort to ensure comparable PICUs, PICU-noGRV delivered continuous feeds over a 24-h period and PICU-GRV-administered bolus feeds more commonly; this may have impacted on the percentage of energy requirements delivered and on feeding tolerance. A further limitation is neither unit uses indirect calorimetry to estimate energy requirements, so energy requirements are based on predictive equations which may be inaccurate [32, 34]. Despite these limitations, we believe that most practices in the two units were similar except for the measurement of GRV: the earlier initiation of feeding in PICU-GRV and the use of continuous feeding in PICU-noGRV. Therefore, this pilot study provides some evidence that not measuring GRV does not increase adverse events or cause harm; however, the impact on energy delivery needs to be examined in a larger multicentre study.

## Conclusions

Routine gastric residual volume measurement is common practice in PICUs internationally. Although we did not demonstrate that this practice significantly impaired the achievement of predicted energy targets in the first 4 days of a child’s PICU admission, consistent achievement of energy targets was higher in PICU-noGRV. Most importantly, however, we found that not measuring GRV did not increase the incidence of vomiting, ventilator acquired pneumonia or necrotising enterocolitis. This is the key concern for clinicians at the bedside. The routine practice of GRV measurement remains important and needs to be questioned, and larger studies are needed in critically ill children to determine the impact on energy targets.

## Abbreviations

CDC: Centre for Disease Control
ETT: Endotracheal tube
EN: Enteral nutrition
GRV: Gastric residual volume
IRB: Institutional Review Board
NEC: Necrotising enterocolitis
PICU: Paediatric Intensive Care Unit
PIM score: Paediatric Index of Mortality
RDA: Recommended dietary allowance
SPSS: Statistical Package for the Social Sciences
VAP: Ventilator-acquired pneumonia
UK: United Kingdom

## References

[1] Ahmad S, Kaitha S, Morton J, Ali T (2012). Nasogastric tube feedings and gastric residual volume: a regional survey. Southern Med J.

[2] Bartlett-Ellis R, Fuehne J (2015). Examination of accuracy in the assessment of gastric residual volume: a simulated, controlled study. JPEN J Parenter Enteral Nutr.

[3] Bockenkamp B, Jouvet P, Arsenault V, Beausejour M, Pelletier V (2009). Assessment of calories prescribed and delivered in critically ill children. Clin Nutr ESPEN.

[4] Bollineni D, Minocha A (2011). Nursing practice of checking gastric residual volumes based on old dogmas: opportunity to improve patient care while decreasing healthcare costs. J La State Med Soc.

[5] Briassoulis G, Zavras N, Hatzis T (2001). Effectiveness and safety of a protocol for promotion of early intragastric feeding in critically ill children. Pediatr Crit Care Med.

[6] Briassoulis G, Briassouli E, Tavladaki T, Ilia S, Fitrolaki DM, Spanaki AM (2014). Unpredictable combination of metabolic and feeding patterns in malnourished critically ill children: the malnutrition–energy assessment question. Intensive Care Med.

[7] Briassoulis G, Ilia S, Meyer R (2016). Enteral nutrition in PICUs: missing not impossible!. Ped Crit Care Med.

[8] Costa C, Tonial C, Garcia P (2016) Association between nutritional status and outcomes in critically-ill pediatric patients—a systematic review. J Pediatr:223–229 10.1016/j.jped.2015.09.005 26854736

[9] Elke G, Felbinger T, Heyland D (2015). Gastric residual volume in critically ill patients: a dead marker or still alive?. Nutr Clin Practi.

[10] Gregory K, Connolly T (2012). Enteral feeding practices in the NICU. Adv Neonatal Care.

[11] Grippa R, Silva P, Barbosa E, Bresolin N, Mehta N, Moreno Y (2017). Nutritional status as a predictor of duration of mechanical ventilation in critically ill children. Nutr J.

[12] Gupta S, Boville B, Blanton R, Lukasiewicz G, Wincek J, Bai C, Forbes M (2015). A multicentred prospective analysis of diagnosis, risk factors, and outcomes associated with pediatric ventilator-associated pneumonia. Pediatr Crit Care Med.

[13] Jotterand-Chaparro C, Taffe P, Moullet C, Depeyre J, Longchamps D, Perez M, Cotting J (2017) Performance of predictive equations specifically developed to estimate resting energy expenditure in ventilated critically ill children. J Pediatr. 10.1016/j.jpeds.2016.12.063 284 10.1016/j.jpeds.2016.12.063 28108105

[14] Kerklaan D, Fivez T, Mehta N, Messoten D, van Rosmalen J, Hulst J, Van den Berghe G, Joosten K, Verbruggen S (2016). Worldwide Survey of Nutritional Practices in PICUs. Pediatr Crit Care Med.

[15] Kuppinger D, Rittler P, Hartl W, Ruttinger D (2013) Use of gastric residual volume to guide enteral nutrition in critically ill patients: a brief systematic review of clinical studies. Nutr J:1075–1079 10.1016/j.nut.2013.01.025 23756283

[16] Leong A, Cartwright K, Guerra C, Joffe A, Mazurak V, Larsen B (2013). A Canadian survey of perceived barriers to initiation and continuation of enteral feeding in PICUs. Pediatr Crit Care Med.

[17] Li YL, Lin HC, Mugas Torrazza R, Parker L, Talaga E, Neu J (2014). Gastric residual evaluation in preterm neonates: a useful monitoring technique or a hindrance?. Pediatr Neonatol.

[18] Martinez E, Pereira L, Gura K, Stenquist N, Ariagno K, Nurko S, Mehta N (2017) Gastric emptying in critically ill children. JPEN J Parenter Enteral Nutr. 10.1177/0148607116686330 10.1177/0148607116686330 28061320

[19] McClave S, Snider H (2002). Clinical use of gastric residual volumes as a monitor for patients on enteral tube feeding. JPEN J Parenter Enteral Nutr.

[20] McClave S, Lukan J, Steafater C, Lowen C, Looney S, Matheson P, Gleeson K, Spain D (2005). Poor validity of residual volumes as a marker for risk of aspiration in critically ill patients. Crit Care Med.

[21] Mehta N, Compher C (2009). A.S.P.E.N. Clinical guidelines: nutrition support of the critically ill child. J JPEN J Parenter Enteral Nutr.

[22] Mehta N, McAleer D, Hamilton S, Naples E, Leavitt K, Mitchell P, Duggan C (2010). Challenges to optimal enteral nutrition in a multidisciplinary pediatric intensive care unit. JPEN J Parenter Enteral Nutr.

[23] Mehta N, Bechard L, Cahill N, Wang M, Day A, Duggan C, Heyland D (2012). Nutritional practices and their relationship to clinical outcomes in critically ill children—an international multicentre cohort study. Crit Care Med.

[24] Montejo J, Minambres E, Bordeje L, Mesejo A, Acosta J, Heras A, Ferre M, Fernandez-Ortega F, Vaquerizo C, Manzandeo R (2010). Gastric residual volume during enteral nutrition in ICU patients: the REGANE study. Intensive Care Med.

[25] Ozen N, Tosun N, Yamanel L, Altintas N, Kilciler G, Ozen V (2016). Evaluation of the effect on patient parameters of not monitoring gastric residual volume in intensive care patients on a mechanical ventilator receiving enteral nutrition: a randomized clinical trial. J Crit Care.

[26] Parker L, Torrazza RM, Yuefeng L, Talaga E, Shuster J, Neu J (2015). Aspiration and evaluation of gastric residuals in the NICU: state of the science. J Perinat Neonatal Nurs.

[27] Poulard F, Dimet J, Martin-Lefevre L, Bontemops F, Fiancette M, Clementi E, Lebert C, Renard B, Reignier J (2010). Impact of not measuring residual gastric volume in mechanically ventilated patients receiving early enteral feeding: a prospective before-after study. JPEN J Parenter Enteral Nutr.

[28] Quenot JP, Plantefeve G, Baudel JL, Camilatto I, Bertholet E, Cailliod R, Reignier J, Rigaud JP (2010). Bedside adherence to clinical practice guidelines for enteral nutrition in critically ill patients receiving mechanical ventilation: a prospective, multicentre, observational study. Criti Care.

[29] Reignier J, Mercier E, Le Gouge A, Boulain T, Desachy A, Bellec F, Clavel M, Frat JP, Quenot JP (2013). Lascarrou JB; for the clinical research in intensive care and sepsis (CRICS) group effect of not monitoring residual gastric volume on risk of ventilator-associated pneumonia in adults receiving mechanical ventilation and early enteral feeding. JAMA.

[30] Ridley E, Davies A (2011) Practicalities of nutrition support in the intensive care unit: the usefulness of gastric residual volume and Prokinetic agents with enteral nutrition. Nutr J:1–4 10.1016/j.nut.2010.10.010 21295944

[31] Schofield W (1985). Predicting basal metabolic rate, new standards and review of previous work. Hum Nutr Clin Nutr.

[32] Tavladaki T, Spanaki AM, Dimitriou H, Briassoulis G (2017) Alterations in metabolic patterns in critically ill patients—is there need of action? Eur J Clin Nutr:431–433 10.1038/ejcn.2016.278 28176774

[33] Tume L, Carter B, Latten L (2013). A UK and Irish survey of enteral nutrition practices in paediatric intensive care units. Br J Nutr.

[34] Tume LN, Latten L, Kenworthy L (2017) Paediatric intensive care nurses’ decision-making around gastric residual volume measurement. Nurs Crit Care. 10.1111/nicc.12304 10.1111/nicc.12304 28640510

[35] Valla F, Gaillard-Le Roux B, Ford-Chessel C et al (2016) NutriRea-Ped 2014: the Nursing survey on nutrition practices in French-speaking pediatric intensive care units. JPGN J Parenter Enteral Nutr 62;2: 174–179. 226 doi:10.1097/MPG.000000000000930 227 10.1097/MPG.0000000000000930 26237373

